# Temperature Chaos, Memory Effect, and Domain Fluctuations in the Spiral Antiferromagnet Dy

**DOI:** 10.1038/s41598-019-41566-7

**Published:** 2019-03-25

**Authors:** Sergey Kustov, Iuliia Liubimova, Miguel Corró, Joan Torrens-Serra, Xiebin Wang, Charles R. S. Haines, Ekhard K. H. Salje

**Affiliations:** 1 Department of Physics, University of Balearic Islands, Cra. Valldemossa km 7.5, Palma de Mallorca, E07122 Spain; 2 ITMO University, 49 Kronverkskiy av., St Petersburg, 197101 Russia; 3 Key Laboratory for Liquid-Solid Structural Evolution and Processing of Materials, Shandong University, Jingshi Road 17923, Jinan, 250061 China; 4 Department of Earth Sciences, University of Cambridge, Cambridge, CB2 3EQ England; 5 Xi An Jiao Tong University, State Key Laboratory for Mechanical Behaviour of Materials, Xian, 710049 Shaanxi China

**Keywords:** Magnetic properties and materials, Condensed-matter physics

## Abstract

The spiral antiferromagnetic phase of polycrystalline dysprosium between 140 K and the Néel temperature at 178 K and its domain wall (DW) dynamics were investigated using high-resolution ultrasonic spectroscopy. Two kinetic processes of quasi-static DW motion occur under non-isothermal and isothermal conditions. A “fast” process is proportional to the rate of the temperature change and results in a new category of anelastic phenomena: magnetic transient ultrasonic internal friction (IF). This IF, related to fast moving magnetic DWs, decays rapidly after interruptions of cooling/heating cycles. A second, “slow” kinetic process is seen as logarithmic IF relaxation under isothermal conditions. This second process is glass-like and results in memory and temperature chaos effects. Low-frequency thermal fluctuations of DWs, previously detected by X-ray photon correlation spectroscopy, are related to critical fluctuations with Brownian motion-like dynamics of DWs.

## Introduction

Anti-ferroic crystals, such as anti-ferromagnets, anti-ferroelectrics, and anti-ferroelastics, form a class of materials that has gained prominence for its unusual domain wall (DW) dynamics^1–7^. The DWs are topologically akin to stacking faults, in contrast to twin boundaries and magnetic DWs in ferroic materials. Anti-ferroic DW energies are often much lower than ferroic domain boundaries^8^ so that the ability to form dense domain patterns is enhanced. This is a key aspect for the investigation of functional domain structures^9^ with emerging DW properties. It is equally a defining element of domain boundary glasses where very high densities of DWs form non-ergodic structural states akin to spin glasses^10,11^. Glass states can be related to jamming of domain boundaries^12–15^. Rare earth dysprosium is a good model system for studying magnetically ordered phases with well characterised structural and magnetic states. It also has a complex temperature –field phase diagram with several ferromagnetic (collinear, angular) and antiferromagnetic (helical, fan, vortex) phases, see e.g.^16^. The dynamics of DWs in antiferromagnets is much more difficult to access than in ferromagnets, since DW motion does not produce magnetic flux changes. Time series of X-ray photon correlation spectroscopy speckle patterns^17,18^ were used to study low-frequency DW dynamics in the antiferromagnetic helical phase of 500-nm thick epitaxial Dy layers^19^. Slow thermally driven DW fluctuations were reported over a narrow temperature range 177.4–179.5 K near *T*_*N*_ = 178 K^19^. It was claimed that these DW movements were not related to critical spin fluctuations and that jammed DWs froze outside the narrow range of fluctuations, at ca. 10 K below *T*_*N*_^19^. Here we report the results of high resolution investigations of acoustic losses or internal friction (IF), which show that fluctuations seen in^19^ do indeed correspond to critical spin fluctuations in the vicinity of antiferromagnetic (AFM) ordering. We also show time-dependent rearrangements of DW structures at 178 K > *T* > 140 K. We argue that these rearrangements are combined with memory and temperature chaos effects, which are typical for glassy systems. Furthermore, temperature dependencies between *T*_*N*_ and 140 K define a new category of anelastic phenomena, namely the magnetic transient ultrasonic IF, which scales as the time derivative of temperature $dT/dt=\dot{T}$ with a maximum near 166 K. This temperature coincides in polycrystalline Dy with the recently reported Villari point *T*_*VP*_^20,21^, where the reversible inverse magnetostriction changes sign and hence passes through zero.

## Materials and Methods

Internal friction, IF, is defined as the logarithmic decrement $\delta =\frac{{\rm{\Delta }}W}{2W}$, with *W* the maximum stored elastic energy and ${\rm{\Delta }}W$ the energy dissipated in a cycle of oscillations. The decrement and the Young’s modulus, *E*, were measured by piezoelectric ultrasonic composite oscillator technique^22^ over a temperature range 80–210 K at frequencies near 90 kHz, which is determined by the type of the quartz transducer used. The frequency range close to 10^5^ Hz corresponds (at least in ferromagnets) to the highest sensitivity to the local displacements of magnetic DWs, producing micro-eddy current damping^23^. Temperature spectra of the internal friction and Young´s modulus were taken either in a continuous mode (uninterrupted scans) or in interrupted mode with isothermal holdings of 40 min. at selected temperatures with consequent resuming of the cooling/heating scan. The oscillatory strain amplitude was stabilized in all experiments at ${\varepsilon }_{0}={10}^{-5}$, sufficiently low not to produce detectable non-linear effects. Dy samples of 13 × 2 × 1 mm^3^ measured half the ultrasonic wavelength and were excited by a standing wave. Two series of samples of different origins were tested. The samples from the first series were spark-cut from 1 mm thick rolled plate, supplied by Sigma Aldrich (99.9% purity). The second series (as cast samples) was cut from an ingot produced by arc melting from 99.0% purity (99.9% purity with respect to rare earth elements) raw material purchased from Suzhou Chemical Co, China. Samples from the first series were studied in as received (rolled) state and after vacuum annealing at 920 K for 2 hours. Samples of all types and heat treatments showed essentially similar results. Annealing of rolled samples eliminated minor differences with as cast samples. If not specified otherwise, the data are presented for the as-cast samples.

## Results

**Figure 1:**
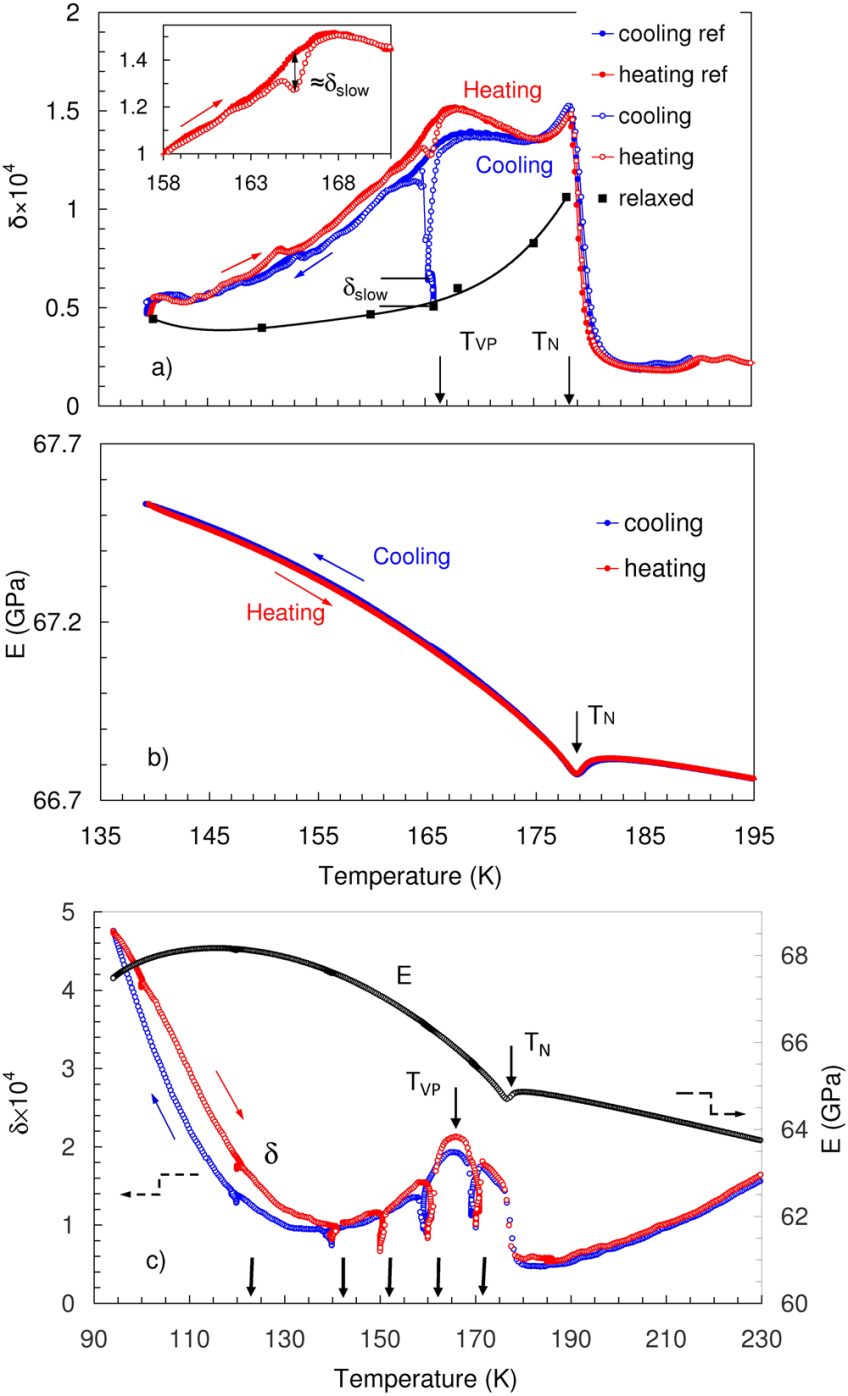
(**a**) Internal friction, δ, in two consecutive thermal cycles 210 K–140 K with a cooling-heating rate of 1 K/min. The first cooling scan was interrupted for 2400 s at 166 K, resulting in a significant decrease of the internal friction level. The reference curves correspond to the next uninterrupted thermal cycle. Black squares are final values of the internal friction reached after holding the following temperatures stable for 2400 s: 140, 150, 160, 166, 168, 175 and 178 K; the line is a guide to the eye. Vertical arrows indicate the Néel temperature T_N_ = 178 K and the Villari point at T_VP_ = 166 K. Symbol ${\delta }_{slow}$ represents the isothermally relaxed component of the internal friction at 166 K (see text). The inset shows details of the memory effect on heating, provoked by isothermal holding on cooling and compares the magnitude of the memory effect with isothermally relaxed internal friction, ${\delta }_{slow}$. (**b**) Temperature spectra of Young’s modulus on cooling and heating for as cast sample. (**c**) Internal friction, *δ*, and Young’s modulus, *E*, in a thermocycle 230-95 K (cooling-heating rate 2 K/min) for as received (rolled) sample. Thermal cycling was interrupted for 2400 s during cooling and heating at temperatures of 170, 160, 150, 140 and 120 K, indicated by vertical arrows.

Figure 1 shows the IF (a) and Young’s modulus (b) between 195 and 140 K for an as-cast sample. A Young’s modulus minimum concomitant with an IF peak at T_N_ = 178 K is typical for an AFM transition^24,25^. The IF spectra were registered under constant cooling/heating rate and differ notably below *T*_*N*_ from the IF behaviour under isothermal conditions $dT/dt=0$^24,25^. The difference is that the IF below *T*_*N*_ does not fall off rapidly for $dT/dt\ne 0$ but remains at a similar level as the absorption peak at *T*_*N*_. Broad maxima of the IF below *T*_*N*_ in Dy for longitudinal and especially for transverse waves were observed under non-isothermal conditions in^26^ but were not commented upon. Strikingly, the non-isothermal IF decreases dramatically, (e.g. by a factor of 3 at 166 K) after interrupting a cooling run and subsequent isothermal relaxation (Fig. 1(a)). The relaxed IF spectrum in Fig. 1(a) (shown by the black line) corresponds to the classical IF pattern in Dy under isothermal conditions^24^. The magnitude of relaxation during isothermal dwelling shows a maximum near the Villari point at 166 K^20,21^.

After resuming the interrupted cooling run the IF reaches rapidly the previous level of continuous cooling. The IF spectrum, registered under consequent uninterrupted heating, shows a local IF dip at the temperature of the isothermal relaxation during cooling. This is the memory effect, exemplified by the data at 166 K in Fig. 1(a), see the inset in Fig. 1(a) for details. The memory effect is determined as a difference between the IF values in an uninterrupted scan and in the local minimum at 166 K, the inset in Fig. 1(a). The memory effect corresponds to the part of the overall IF relaxation, which is represented by its isothermal component ${\delta }_{slow}$ (inset in Fig. 1(a)). Heating the sample above *T*_*N*_ erases this memory effect (Fig. 1(a)): it does not reappear in a consecutive uninterrupted cycle. Figure 1(c) depicts similar results for as received (rolled) sample. Several minor differences with the data for as-cast samples, Fig. 1(a,b), can be noted. First, the IF above *T*_*N*_ is higher in rolled sample and the peak at *T*_*N*_ cannot be clearly discerned, presumably due to the high density of defects (dislocations). Second, a minor difference in the YM values of the two samples can likely be accounted for by different textures. For the as received (rolled) sample, the lowest temperature of the thermal cycle was 95 K and cooling/heating scans were interrupted at several temperatures: 170, 160, 150, 140 and 120 K, showing the same overall IF trend and the same relaxation on cooling and heating. Thus, the relaxation is a generic effect in helical AFM polycrystalline dysprosium, which persists in samples of different origin and thermomechanical history. The relaxation process observed on cooling/heating depends on the cooling/heating rate just before the interruption, but is not sensitive to the previous history of the sample (previous interruptions or variations of the cooling rate) as shows Fig. 1(c). This feature is a hallmark of ‘temperature chaos’^27,28^ and, together with the memory effect, describes a glassy state^27–29^.

**Figure 2:**
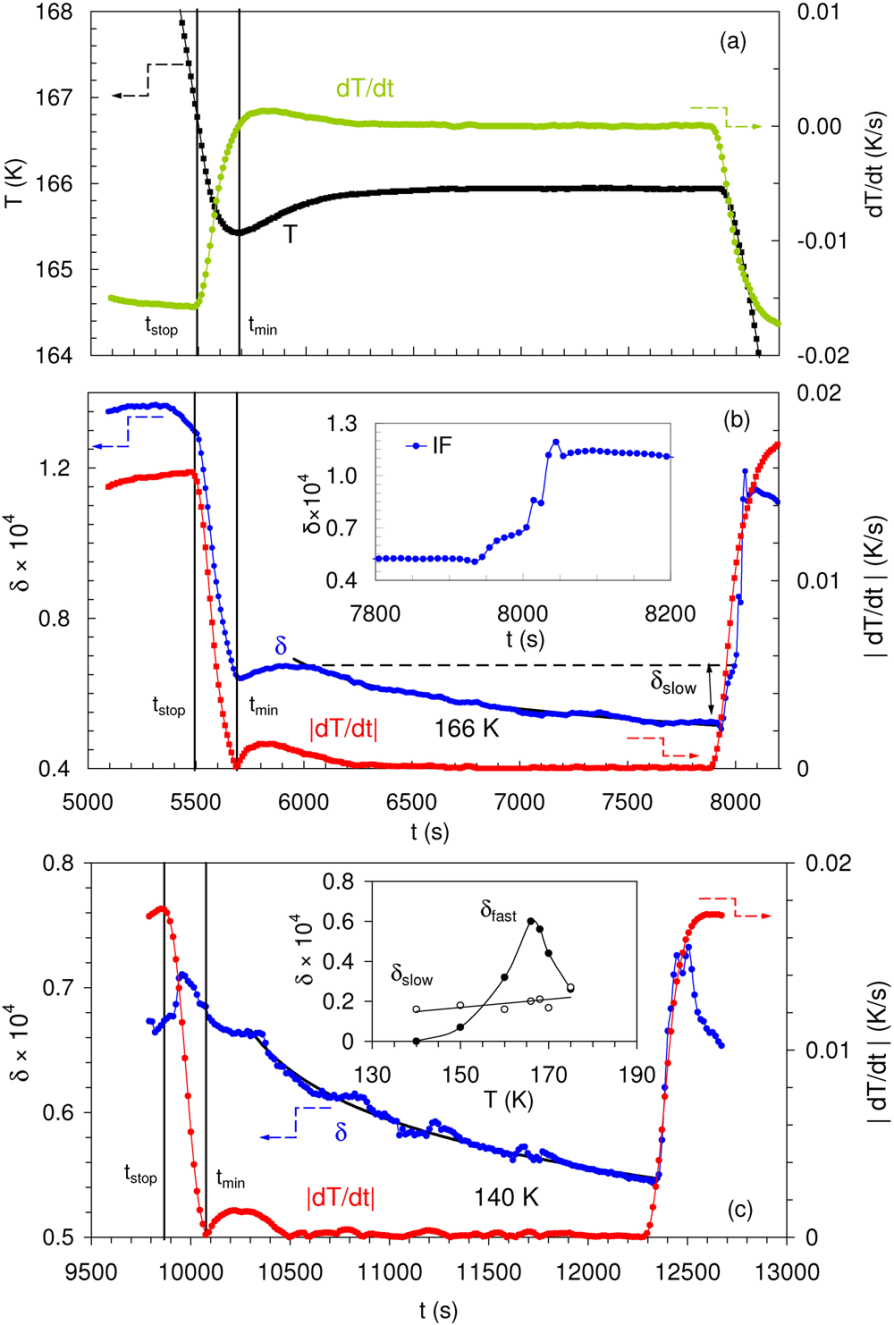
(**a**) Time dependences of temperature *T* and cooling rate $\,dT/dt$ and (**b**) of internal friction *δ* and absolute value of the cooling rate $|dT/dt|$ during interruption of cooling at 166 K; (**c**) same dependences as in (**b**) during interruption of cooling at 140 K. The inset in (**b**) shows on an expanded time scale the kinetics of the internal friction during restart of interrupted cooling. Vertical lines mark *t*_*stop*_ as the time when the temperature ramping is interrupted and *t*_*min*_ is the time when the temperature is at a minimum during thermal overshoot. ${\delta }_{slow}$ in (**b**) denotes isothermal relaxation during 2400 s (defined later in Fig. 3). The inset in (**c**) shows temperature dependences of ${\delta }_{slow}$ and the fast relaxation component, ${\delta }_{fast}$. Data obtained during several temperature scans interrupted at different temperatures are shown. The lines are guides to the eye. Fitting of the internal friction kinetics with logarithmic functions are shown in (**b**,**c**).

**Figure 3:**
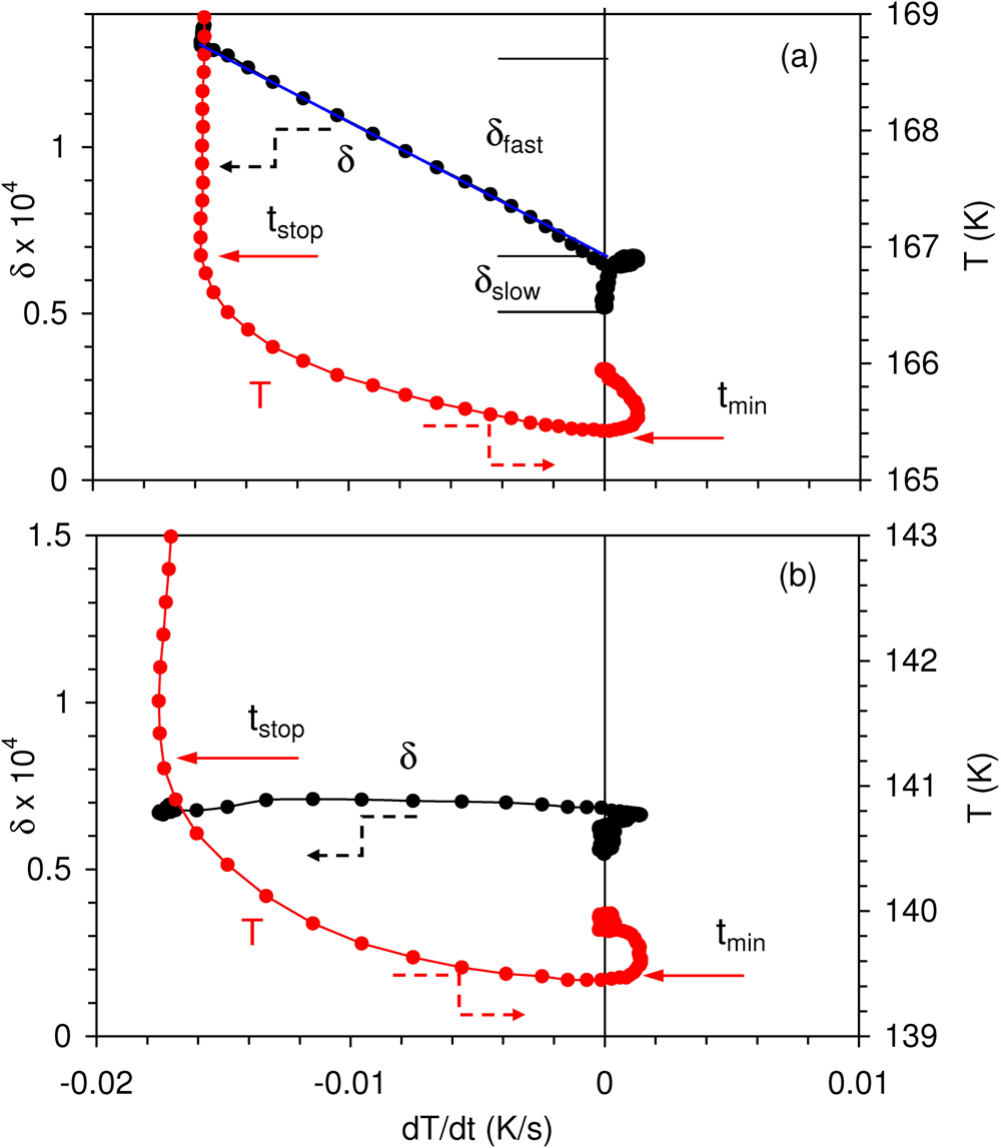
Internal friction $\delta$, and temperature *T* versus temperature rate $dT/dt$ during interruptions of cooling near 166 K (**a**) and 140 K (**b**). Proportionality between $\delta$ and $|\dot{T}|$ is seen near 166 K (black curve in (**a**)) but not at 140 K. ${\delta }_{slow}$ in (**a**) stands for the estimation of the “slow” isothermal relaxation, Eq. (1), and is shown also in Figs 1(a), and 2(b). ${\delta }_{fast}$ in (**a**) represents the “fast” component of the internal friction, Eq. (1). ${\delta }_{slow}$ and ${\delta }_{fast}$ versus temperature are shown in the inset in Fig. 2(c). ${t}_{stop}$ and ${t}_{\min }$ in (**a**,**b**) are the same as in Fig. 2(a) for the temperature of 166 K, and Fig. 2(c) for the temperature of 140 K.

Figure 2 shows the IF relaxation during interruptions of cooling at 166 K (a,b) and 140 K (c). Time dependences of the temperature *T* and cooling rate $\dot{T}=dT/dt$ during interruption of cooling at 166 K are shown in Fig. 2(a), corresponding variations of the IF are compared with the absolute value of cooling rate $|dT/dt|$ in Fig. 2(b). Figure 2(c) depicts the IF kinetics for interruption of cooling at 140 K. Two distinct dynamic processes are found in IF kinetics: firstly a slow relaxation when the temperature is constant ($\dot{T}=0$) and, secondly, a component $\delta  \sim |\dot{T}|$ detected during temperature stabilization. The latter is dominant in the IF kinetics after interruption of cooling at 166 K but is almost absent at 140 K. The IF and temperature data from Fig. 2(b,c) are re-plotted in Fig. 3(a,b) as function of $\dot{T}$ and confirm a strong decrease of the IF proportional to $\dot{T}$ during interruption of cooling at 166 K (a) and its absence at 140 K (b). The proportionality between $|\dot{T}|$ and the IF extends over a large interval of negative values of $\dot{T}$ and is kept for positive $\dot{T}$ during heating from the small negative overshoot (Fig. 3(a)). The proportionality to $|\dot{T}|$ creates an IF minimum in Fig. 2(b) near 5700 s. Thus, the overall IF is a function of temperature, *T*, time *t* (seen as “slow” isothermal relaxation component), and cooling/heating rate $|\dot{T}|$ (responsible for the “fast” relaxation). In a simplest form, assuming independence of slow, ${\delta }_{slow}$, and fast, ${\delta }_{fast}$, IF components, the overall IF $\delta (T,t,|\dot{T}|)$ is:

1 $$\delta (T,t,|\dot{T}|)={\delta }_{0}(T)+{\delta }_{slow}(T,t)+{\delta }_{fast}(T,|\dot{T}|),$$

where ${\delta }_{0}(T)$ is the temperature spectrum of fully relaxed IF. The black line in Fig. 1(a) is an approximation to such a spectrum obtained after 2400 s of relaxation.

The isothermal IF relaxation component, ${\delta }_{slow}$ during complete stoppage at 166 K is estimated in Fig. 3(a) as a distance between the line $\delta (\dot{T})$ at $\dot{T}=0$ and the lowest IF value achieved during relaxation. The crossing point of the linear $\delta (\dot{T})$ dependence with the line $\dot{T}=0$ determines the fast $|\dot{T}|$- dependent term, ${\delta }_{fast}$, Fig. 3(a). ${\delta }_{slow}$ is small by comparison with ${\delta }_{fast}$ at 166 K. ${\delta }_{slow}$ defined from the data of Fig. 3(a) is shown in Figs 2(b) and 1(a). According to Fig. 1(a), the magnitude of the memory effect coincides with the degree of isothermal relaxation. Hence, only this sluggish, isothermal relaxation contributes to the memory effect, but not the $|\dot{T}|$ -dependent kinetics. Figure 2(b) indicates that, during resuming of cooling, the jerky recovery of the steady state IF value occurs once the IF gradually increases to the level observed before the isothermal relaxation, i.e. increases by the same value of ${\delta }_{slow}$. The $|\dot{T}|$ dependence is extremely small at 140 K, Fig. 3(b), so that the only variation in $\delta$ exists at the isothermal point $|\dot{T}|=0$. The inset in Fig. 2(c) shows ${\delta }_{slow}$ and ${\delta }_{fast}$ versus temperature. ${\delta }_{slow}$ gradually declines on cooling, whereas ${\delta }_{fast}$ has a clear maximum at 166 K, the temperature of Villari point in Dy^20,21^.

Figure 2(b,c) shows fittings of the IF sluggish isothermal relaxation at 166 K and 140 K with a logarithmic law. Excluding the initial transitory period of temperature stabilization, the data agree well with logarithmic trends.

## Discussion

The distinguishing feature of the IF spectra of Dy are their low values. The IF increases nearly an order of magnitude upon ordering in the helical AFM phase with $\delta \approx {10}^{-4}$, Fig. 1(a). This value is still small compared with the IF of Dy in the ferromagnetic state^26,30^. The increase of IF in the helical phase compared with the paramagnetic state indicates that anelastic phenomena below *T*_*N*_ originate from an ordered magnetic structure. No resonance phenomena are expected because our measurement frequency 10^5^ Hz is much smaller than relaxation frequencies of individual spins or spin waves. The frequency near 10^5^ Hz corresponds to the maximum sensitivity for collective spin rearrangements represented by DW-related magnetomechanical (microeddy current) IF in ferromagnets^23^. This interpretation of FM domain wall related IF is not directly applicable to pure AFM structures. In helical AFM Dy, however, DWs perpendicular to the c-axis possess a net magnetic moment and their motion can be the origin of DW-related magnetic IF. Therefore, we consider DWs as relevant structure contributing to the IF at ~10^5^ Hz.

The IF under continuous cooling/heating contains two kinetic processes. The first is a sluggish isothermal relaxation with logarithmic kinetics, and the second is a fast $|\dot{T}|$- dependent component. The “sluggish” isothermal relaxation with logarithmic kinetics contributes to the memory effect. It involves ageing of a glassy system, which moves it towards a global energy minimum and reflects the internal restructuring of the magnetic microstructure (DW configurations) at *T* > 140 K. In contrast, our fast relaxation IF component scales linearly with $|\dot{T}|$ over a wide temperature range and is typical in the behaviour of transient IF, ${\delta }_{trans}$. ${\delta }_{trans}$ accompanies macro- and microplastic deformation of crystals^31–33^ and first order structural transitions^34–36^. Non-magnetic ${\delta }_{trans}$ is approximately inversely proportional to the oscillation frequency $\omega$^31–36^:

2 $${\delta }_{trans}\propto {(\frac{1}{\omega })}^{n}$$

with $n <  \sim \,1$. The nearly inverse frequency dependence makes non-magnetic ${\delta }_{trans}$ detectable only at low frequencies (<~10^2^ Hz) and negligible at ultrasonic frequencies^31,36^.

We now discuss possible structural origins of ${\delta }_{trans}$ in polycrystalline Dy. Firstly, the helical AFM state shows negative thermal expansion along the hexagonal axis^37,38^ and positive thermal expansion in the basal plane^37^. Temperature changes are then expected to provoke significant variations of exchange energies and change the pitch of the helical structure^39^. Variations of pitch result in the rotation of the magnetic moments at DWs during temperature changes. The rotation of magnetic moments affects the energy of their dipolar interactions and provokes a continuous rearrangement of the DW structure over a wide temperature range. Secondly, the strong anisotropy of thermal expansion may result in intense thermal stresses in polycrystalline Dy, also affecting DW configurations over the range centred around the Villari point at ca. 166 K. Experiments with single crystals should allow one of the two scenarios to be chosen. Since the motion of DWs in AFM Dy is accompanied by hysteresis^21,40^, the combination of translational and hysteretic oscillatory motion of DWs with magnetic moments may induce eddy currents with ${\delta }_{trans} \sim \omega$ via the Faraday law. This proportionality cancels out the conventional inverse frequency dependence of ${\delta }_{trans}$, Eq. (2), and thus makes the magnetic transient term detectable at ultrasonic frequencies as a fast IF component, proportional to $|\dot{T}|$. The magnetic transitory IF term is expected to be nearly frequency-independent up to a frequency of microeddy current relaxation and fall off rapidly above this frequency limit. For ferromagnets, the frequency of microeddy current relaxation ranges from ca. 10^5^ Hz^23^ to several MHz^41^. Very low IF levels in the AFM Dy do not permit verification of the IF $dT/dt$ dependence at very low frequencies due to poor experimental resolution^30,42,43^. Figure 2(c) and the inset show that the “fast” IF component nearly disappears below approximately 140 K with blocking of the translational motion of DWs. We suggest that the origin of this blocking is the emergence of ferromagnetically ordered nuclei. The following observations support this hypothesis.

- The IF level increases abruptly on cooling below ca. 140 K, Fig. 1(c).
- The IF (and YM) hysteresis emerge just at this temperature, see Fig. 1(c).
- The YM started to soften, Fig. 1(c), as precursor to the ferromagnetic phase^44^.
- The IF is promoted by weak magnetic fields below 140 K (not shown). This effect is consistent with the emerging net magnetization provoking macroeddy current damping.

Ferromagnetic nuclei interact strongly with antiferromagnetic DWs and block efficiently their translational motion while this leaves the possibility of local isothermal rearrangements (represented by ${\delta }_{slow}$).

The motion of DWs and the two kinetic processes are confirmed by two-stage recovery of the steady state IF after resumed cooling. Just after the restart of cooling, the IF increases gradually until the IF level reaches the same value as before the slow relaxation (inset in Fig. 2(b)). During this stage, DWs recover unrelaxed “de-pinned” states without notable large-scale movement and no $|\dot{T}|$ dependency before the IF level reaches the “de-pinned” level. Once the “de-pinned” state is reached, the IF increases abruptly towards the steady state value due to the fast recuperation of the transient IF term, i.e. movements of DWs. Their fast initiation results in jerky behaviour under constant $|\dot{T}|$ and stems from the undercooling required to “de-pin” DWs before they start to move.

**Figure 4:**
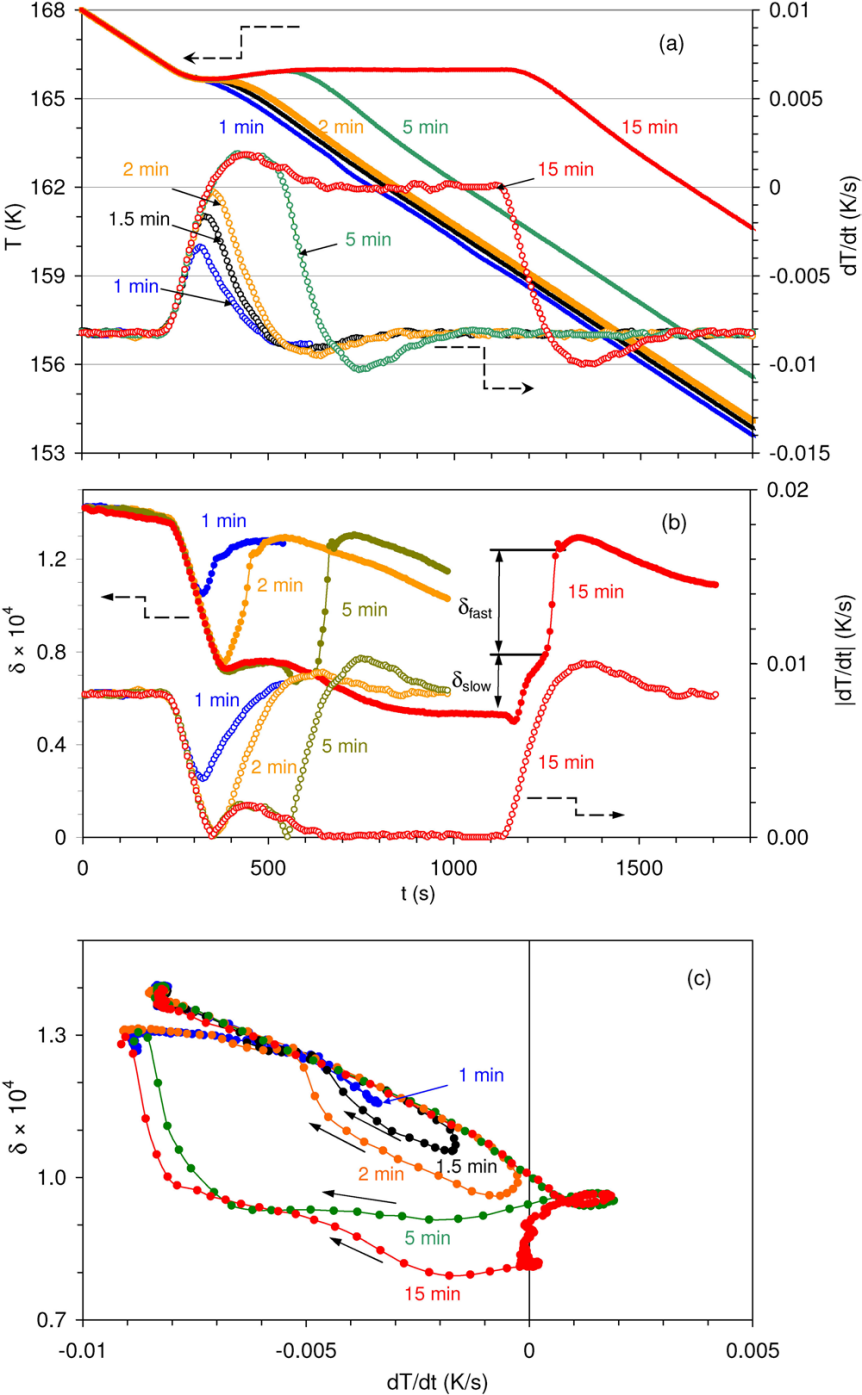
(**a**) Time dependences of temperature *T* (full symbols) and cooling rate $dT/dt$ (open symbols) during interruptions of cooling (0.5 K/min), isothermal holdings of the sample at 166 K programmed for 1, 1.5, 2, 5 and 15 min and resuming of cooling; the origin of the time scale, $t=0$, is taken for all experiments at 168 K; (**b**) Time dependences of the absolute values of cooling rate $|dT/dt|$ taken from (**a**) (open symbols), and of internal friction $\delta$ (full symbols) during interruptions of cooling scans at 166 K programmed for 1, 2, 5 and 15 min and resuming of cooling; slow and fast components of internal friction, ${\delta }_{slow}$ and ${\delta }_{fast}$, respectively, as revealed during resuming of cooling, are indicated; (**c**) internal friction $\delta$ versus cooling rate $dT/dt$ during interruptions and resuming of cooling shown in panel (**a**); black arrows indicate variation of the internal friction during resuming of cooling.

In order to additionally confirm the existence and hierarchy of the two steps in the recovery of the steady-state DW dynamics, experiments with variable time of isothermal dwelling were performed. Figure 4 shows the temperature and $dT/dt$ versus time (a), the absolute value of $dT/dt$ and the IF versus time (b), and IF versus $dT/dt$ (c) during several interruptions of cooling at 166 K. Isothermal segments from 1 to 15 min were pre-set. The shortest isothermal segment (1 min) results in a brief decrease of the cooling rate: the system does not reach the pre-set isothermal dwelling. Under these circumstances, the IF changes reversibly and is essentially proportional to $\dot{T}$, Fig. 4(b). The increase of the duration of the isothermal segment provokes a time-dependent decrease of the IF level (data in Fig. 4(b)) and the emergence of the IF hysteresis versus $dT/dt$, Fig. 4(c). For longer dwelling times, the separation of the IF into the slow and fast components becomes even more evident, Fig. 4(b). During renewed cooling the “fast” IF increases rapidly (in a jerky manner with a small overshoot, Fig. 4(b)) to the steady state level after undercooling by ca. 0.5 K. This tendency confirms that the occurrence of the sluggish relaxation progressively impedes transient IF, associated with the motion of DWs.

Finally, the concept of low-frequency DW thermal fluctuations in Dy^19^ is revisited. DW speckle variations were reported over a narrow temperature range 179.4–177.5 K^19^, reflecting low-frequency thermal DW fluctuations. Critical spin fluctuations were discarded as a possible origin of these fluctuations, due to a strong difference in the characteristic frequencies of critical fluctuations and time/frequency window of experimental observations^19^. The relaxation time of the spin system diverges approaching the critical point. The critical slowdown is represented by the critical ultrasonic attenuation peak at *T*_*N*_^25^. The temperature range of the critical attenuation (ca. 175–180 K, Fig. 1(a) and ref.^25^). coincides with the range (179.4–177.5 K) where long-term variations of the speckle patterns were reported in^19^. Fluctuations may induce Brownian motion of DWs, which can be detected on a long time scale. Thus, low-frequency fluctuations of DWs in Dy close and just below the *T*_*N*_ are related to critical fluctuations. Moreover, the absence of DW speckle variations outside the range of critical fluctuations does not mean that the DWs are frozen some 10 K below *T*_*N*_^19^. Our observations of magnetic transient IF well below *T*_*N*_ (to approx. 150 K) and of the IF relaxation at lower temperatures indicate DW motion down to ca. 140 K.

Our observations of the slow DW dynamics in AFM Dy are consistent with an overall picture in various ferroics and multiferroics. Slow kinetics movements have been observed in antiferroelectric materials^45^ and wall meandering and slow relaxations at high temperatures are dominant in LiNbO_3_ where kinks in walls occur at very high temperatures^46^. When walls intersect in LaAlO_3_, they form tweed structures which also remain (meta-)stable unless heated above the ferroelastic transition point. This tweed is locally dipolar and the polarity persists again if the sample has not been heated^47^. Tweed structures are just one example of domain glass states^10,11^, which are quasi stable with relaxations towards the uniform equilibrium state being so slow that no macroscopic relaxation has yet been observed experimentally. Finally, extremely slow relaxations are predicted to occur in all jammed ferroelastic materials, such as SrTiO_3_ where inter-boundary relaxations and jamming prevent fast kinetic processes towards equilibrium and where sluggish relaxations dominate at sufficiently low temperatures^48^.

As for the second, “fast” IF component or transient ultrasonic IF of magnetic origin, we predict its existence in ferromagnets and multiferroics under different experimental conditions, implying rearrangement of the magnetic DW structure. In particular, magnetic transient ultrasonic IF should exist, during temperature variations, in such multiferroics as ferromagnetic martensites. The microplastic straining of anisotropic martensitic variants through the displacement of twin boundaries^32^ provokes the “mechanical” transitory IF which is negligible at ultrasonic frequencies^31,36^, Eq. (2). Rearrangement of magnetic DWs associated with twin boundaries motion under thermal stresses together with their oscillatory motion are expected to be mediated by magnetoelastic coupling and can lead to the transitory IF of magnetic origin. Another possible scenario of observations of magnetic transitory ultrasonic IF is the formation of tweed structure in ferromagnets, like premartensitic transition in Ni_2_MnGa^49,50^. Experimental studies of these predicted phenomena are ongoing.

## Summary and Conclusions

- We observe in antiferromagnetic Dy a new category of anelastic phenomena - ultrasonic transitory internal friction, related to the fast rearrangement of domain wall structure - and predict its existence in other ferroics and multiferroics under different conditions. In polycrystalline Dy the maximum intensity of the transitory term is centered around Villari point at ca. 166 K.
- The fast rearrangement of the domain wall structure is followed by the isothermal relaxation with logarithmic kinetics, which demonstrates features typical in glassy systems: temperature chaos and memory effects.
- Low-frequency thermal fluctuations of DWs, previously detected by X-ray photon correlation spectroscopy close to the Néel temperature, are related to critical fluctuations with Brownian motion-like dynamics of DWs.
