# Author Correction: Temperature Chaos, Memory Effect, and Domain Fluctuations in the Spiral Antiferromagnet Dy

**DOI:** 10.1038/s41598-019-46782-9

**Published:** 2019-07-24

**Authors:** Sergey Kustov, Iuliia Liubimova, Miguel Corró, Joan Torrens-Serra, Xiebin Wang, Charles R. S. Haines, Ekhard K. H. Salje

**Affiliations:** 1 Department of Physics, University of Balearic Islands, Cra. Valldemossa km 7.5, Palma de Mallorca, E07122 Spain; 2 ITMO University, 49 Kronverkskiy av., St Petersburg, 197101 Russia; 3 Key Laboratory for Liquid-Solid Structural Evolution and Processing of Materials, Shandong University, Jingshi Road 17923, Jinan, 250061 China; 4 Department of Earth Sciences, University of Cambridge, Cambridge, CB2 3EQ England; 5 Xi An Jiao Tong University, State Key Laboratory for Mechanical Behaviour of Materials, Xian, 710049 Shaanxi China

Correction to: *Scientific Reports* 10.1038/s41598-019-41566-7, published online 25 March 2019

The Acknowledgements section in this Article is incorrect.

“The work was supported by the Spanish Ministerio de Economía y Competitividad, ProjectMAT2014-56116-C04-01-R and by the Ministry of Education and Science of the Russian Federation, goszadanie No. 3.1421.2017/4.6. EKHS is grateful to EPSRC (EP/P024904/1) and the Leverhulme trust (EM-2016-004).”

Should read:

“The work was supported by FEDER/Ministerio de Ciencia, Innovación y Universidades – Agencia Estatal de Investigación/MAT2014-56116-C04-01-R and by the Ministry of Education and Science of the Russian Federation, goszadanie No. 3.1421.2017/4.6. EKHS is grateful to EPSRC (EP/P024904/1) and the Leverhulme trust (EM-2016-004).”

